# Tight junction stabilization prevents HepaRG cell death in drug-induced intrahepatic cholestasis

**DOI:** 10.1242/bio.058606

**Published:** 2021-06-22

**Authors:** Rie Sonoi, Yoshihisa Hagihara

**Affiliations:** Biomedical Research Institute, National Institute of Advanced Industrial Science and Technology, 1-8-31 Midorigaoka, Ikeda, Osaka 563-8577, Japan

**Keywords:** Bile canaliculi dynamics, Drug-induced intrahepatic cholestasis, HepaRG cells, Tight junction, Zonula occludens-1

## Abstract

Entacapone (ENT), a catechol-*O*-methyltransferase inhibitor, causes liver injury by inducing bile canaliculi (BC) dilation through inhibition of the myosin light kinase pathway. Loss of tight junctions (TJs) induces hepatocyte depolarization, which causes bile secretory failure, leading to liver damage. To understand the influence of TJ structural changes as a consequence of BC dynamics, we compared the datasets of time-lapse and immunofluorescence images for TJ protein ZO-1 in hepatocytes cultured with ENT, forskolin (FOR), ENT/FOR, and those cultured without any drugs. Retrospective analysis revealed that the drastic change in BC behaviors caused TJ disruption and apoptosis in cells cultured with ENT. Exposure to FOR or sodium taurocholate facilitated TJ formation in the cells cultured with ENT and suppressed BC dynamic changes, leading to the inhibition of TJ disruption and apoptosis. Our findings clarify that hepatocyte TJ stabilization protects against cell death induced by BC disruption.

## Abstract (precis)

**Summary:** Exposure to forskolin or sodium taurocholate facilitates tight junction (TJ) formation in HepaRG cells cultured with entacapone and suppresses bile canaliculi dynamic changes, leading to the inhibition of TJ disruption and cell apoptosis.

## INTRODUCTION

Drug-induced liver injuries cause serious adverse events, and intrahepatic cholestasis accounts for approximately 40% of all drug-induced liver injuries (Lee, 2003). On the one hand, many drugs induce intrahepatic cholestasis, inhibit hepatic transporter functions, and result in hepatotoxicity due to the accumulation of bile acids in the cytosol or in the bile canaliculi (BC) of hepatocytes (Pedersen et al., 2013; Qui et al., 2016). On the other hand, drugs that induce intrahepatic cholestasis cause alterations in BC dynamics via the Rho kinase/myosin light kinase pathway (Burbank et al., 2016; Sharanek et al., 2016). Ultimately, bile acids induce mitochondrial impairment and the production of reactive oxygen species in hepatocytes (Rodrigues et al., 2018). Interestingly, some of these drugs do not necessarily inhibit hepatic transporter function (Burbank et al., 2016), and the complicated mechanism underlying intrahepatic cholestasis remains unclear. For example, entacapone (ENT) is clinically used for the treatment of patients with Parkinson's syndrome, but is known to evoke liver injury, such as intrahepatic cholestasis (Fisher et al., 2002), in approximately 0.2% of the patients treated with ENT (Lew and Kricorian, 2007; Haasio , 2010; Grünig et al., 2017).

Defects in tight junction (TJ) proteins, namely occludin and claudin, give rise to depolarization-associated liver injury and cholestasis (Gissen and Arias, 2015; Pradhan-Sundd and Monga, 2019). TJs completely seal BC in hepatocytes, which have polarity and structure (Wood, 1965; Montesano et al., 1975; Luzzatto, 1981). Adaptor proteins such as zonula occludens-1 (ZO-1) and zonula occludens-2 (ZO-2) interact with TJs and the actin cytoskeleton (Itoh et al., 1997; Fanning et al., 1998). These interactions between TJs and the actin cytoskeleton are essential for the maintenance of TJ integrity and polarity, suggesting that the ZO proteins, in addition to the actin cytoskeleton, play a key role in the maintenance of hepatic structures and functions. BC dynamics are regulated by actin filament assembly and disassembly (Burbank et al., 2016; Sharanek et al., 2016). Thus, the relationship between TJ proteins and BC behaviors may make it possible to provide information to help unveil the hidden mechanisms of intrahepatic cholestasis.

Human HepaRG cells are heterogeneous cell populations of hepatocytes and bile epithelial cells (Parent et al., 2004), have the same polarity in accordance with TJ maturation as that of hepatocytes, and exhibit correctly polarized distributions of transport proteins (Andersson et al., 2012). In the present study, we aimed to describe the effect on cells of TJ disruption that occurs as a consequence of BC dynamics in HepaRG cells during drug-induced intrahepatic cholestasis and conducted a retrospective analysis based on time-lapse observations and TJ immunostaining images of HepaRG cells exposed to ENT.

## RESULTS

### Dynamic behavior of BC causes structural changes

ENT, which causes drug-induced intrahepatic cholestasis, decreases myosin light chain 2 phosphorylation through myosin light chain kinase and results in BC dilation (Burbank et al., 2016; Sharanek et al., 2016). Therefore, we used ENT to understand the influence of BC dynamics on TJ structure. A time-lapse analysis was conducted on HepaRG cells cultured without and with ENT (100 μM) at *t*=0–48 h (Movie 1). HepaRG cells at *t*=48 h were fixed and immunofluorescence stained for TJ protein ZO-1, and the images of stained cells were captured in the same region as that observed in the time-lapse observation. Under both culture conditions, HepaRG cells formed the BC between mature hepatocytes. The BC of HepaRG cells cultured without ENT showed gradual repetition of constriction and dilation. However, the HepaRG cells cultured with ENT exhibited rapid dilation of the BC at *t*=0–3 h. With elapsed exposure time, HepaRG cells cultured with ENT showed an increase in the size of BC, followed by their subsequent constriction. By *t*=48 h, the cells with constricted BCs in culture with ENT ultimately led to cell death. Almost all cells in culture without ENT exhibited a smaller alteration in the size of the BC and had a distinct line of ZO-1 that surrounded the BC perfectly. Conversely, almost all cells cultured with ENT were observed to have a drastic alteration in the size of BC and did not express ZO-1 at BC.

**Fig. 1.**
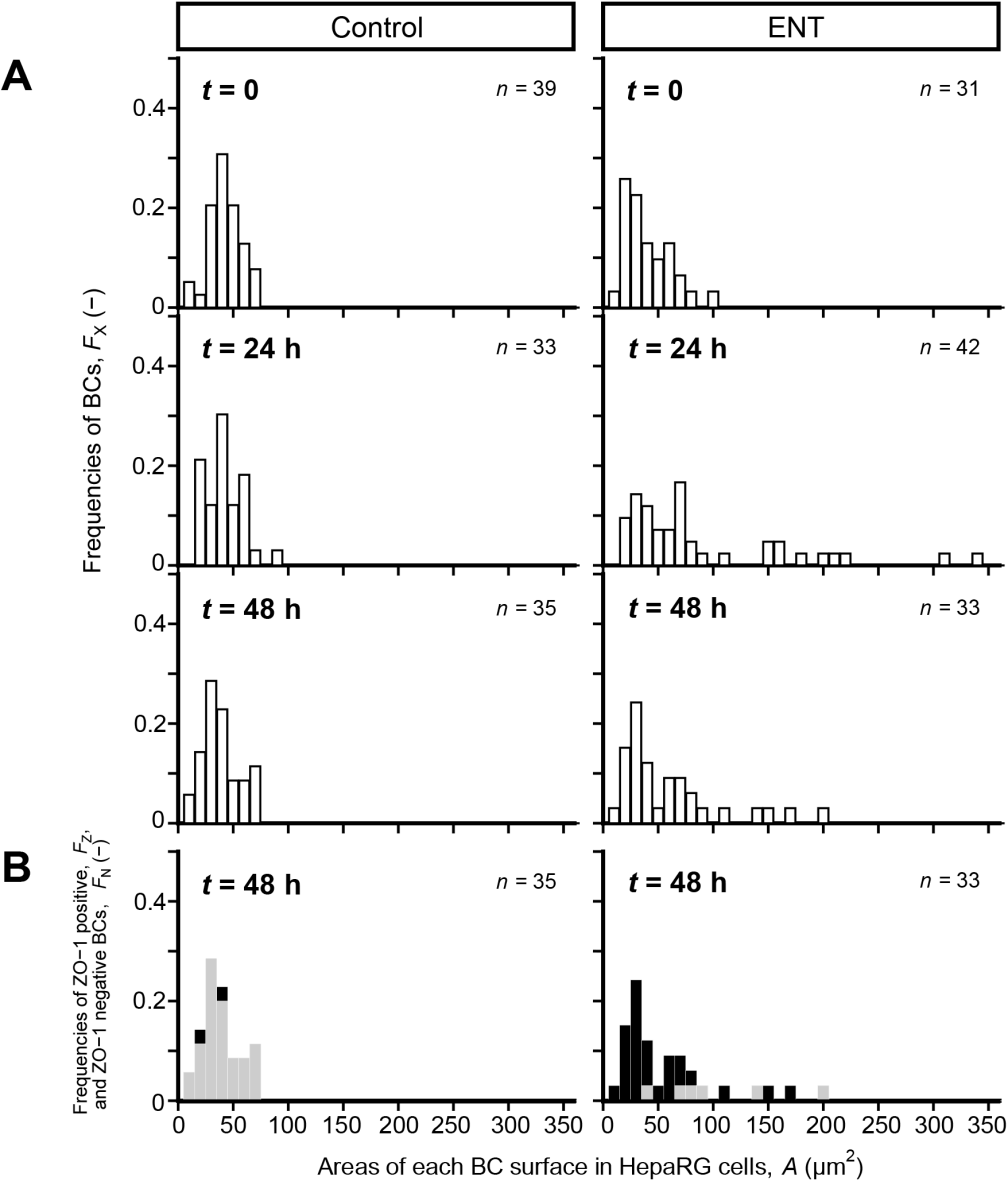
**Size change of BC causes the disruption of TJs in HepaRG cells.** (A) Frequencies of numbers against surface areas of BC of HepaRG cells in cultures with and without ENT (100 µM). Exposure time, *t*, was counted from the moment of the addition of ENT. After time-lapse observation, HepaRG cells were fixed and stained for ZO-1 at *t*=48 h. The surface areas of BC were analyzed based on the data that were obtained from HepaRG cells in the same region where the immunostaining image for ZO-1 was captured (Movie 1 and Fig. S1). (B) Frequencies of ZO-1-positive (*F*_Z_) and ZO-1-negative BC (*F*_Z_) versus surface areas of BC in HepaRG cells at *t*=48 h. The BC of HepaRG cells were divided into two groups, namely, ZO-1-positive and ZO-1-negative BC, respectively. *F*_Z_ (shaded bars) and *F*_N_ (closed bars) values were obtained from HepaRG cells in the region of interest (ROI, 300×300 µm).

To quantitatively understand the change in TJ structure in response to BC dynamics, frequencies of ZO-1-positive (*F*_Z_) or ZO-1-negative BC (*F*_N_) were evaluated. At *t*=0, the average area of BC surfaces in the cells cultured without ENT was 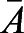
=38±14 μm^2^, and the distribution of *A* ranged from 7.7 to 67 μm^2^ (Fig. 1A). There was no change in the distribution of *A* in the culture without ENT until *t*=48 h (Fig. 1A). However, the trend of distribution of *A* in the culture with ENT at *t*=0 was similar to that in the culture without ENT at *t*=0 (Fig. 1A). With elapsed exposure time, a broad distribution of *A* was observed, ranging from 12 to 330 μm^2^, and the distribution of *A* in the culture with ENT at *t*=48 h was narrower than that at *t*=24 h (Fig. 1A). At *t*=48 h, the *F*_Z_ value in the culture without ENT was 0.94, whereas that in the culture with ENT was 0.18 (Fig. 1B). These results suggest that the drastic change in the size of the BC surface resulted in the disruption of TJ in HepaRG cells.

**Fig. 2.**
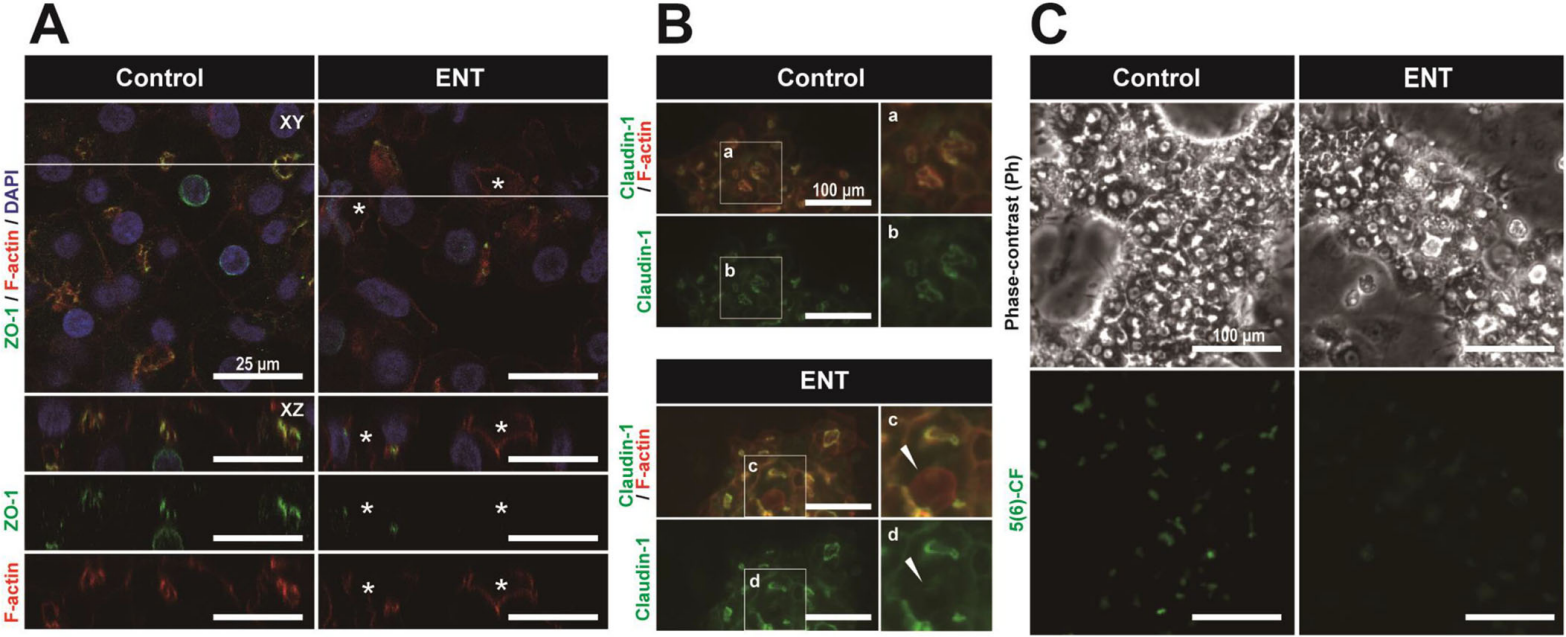
**Exposure of ENT to HepaRG cells alters the structure and function of BC.** (A) Confocal immunofluorescence images of cell nuclei (DAPI, blue), TJ protein (ZO-1, green), and F-actin (Phalloidin, red) for HepaRG cells in culture with and without ENT at *t*=48 h. Confocal immunofluorescence images show two-dimensional optical cross-sectioning (XZ and XY planes). XZ panels (lower three panels) are images of the vertical view of the white lines on horizonal view images. The asterisks indicate the dilated BC. (B) Immunofluorescence images of F-actin (Phalloidin, red) and TJ protein (Claudin-1, green) for HepaRG cells in cultures with and without ENT at *t*=48 h. Solid arrowheads denote the disruption of TJ in dilated BC. (C) The images of phase-contrast and 5(and 6)-carboxyfluorescein [5(6)-CF] in BC of HepaRG cells. To investigate the hepatocyte function, we used 5(and 6)-carboxyfluorescein diacetate [5(6)-CFDA], which is hydrolyzed to 5(6)-CF. 5(6)-CF is secreted from hepatocytes into the BC, accumulates in BC, and exhibits distinctive fluorescence (green). Scale bars, 25 and 100 μm.

To ascertain the influence of BC dynamics on the structure and function of hepatocytes, we stained the cell nuclei, F-actin, TJ protein ZO-1, and claudin-1, and compared their localization at *t*=48 h. Confocal images for ZO-1 and F-actin revealed that ZO-1 expression was observed in BC between neighboring cells achieving maturation and was lost in BC of the cells exhibiting the BC dilation (Fig. 2A). In the culture with ENT, dilation of BC pressed the nuclei of neighboring cells, and the morphology of the cell nucleus showed an elliptical shape (Fig. 2A). F-actin and claudin-1 were colocalized in the cellular membrane of cells in the culture without ENT (Fig. 2B), but claudin-1 was not localized in dilated BC in the culture with ENT (Fig. 2B). To understand hepatocyte function and BC structure, we used 5(and 6)-carboxyfluorescein diacetate [5(6)-CFDA], which produces fluorescence through hydrolysis reaction in hepatocytes. Thus, the accumulation of fluorescent 5(6)-carboxyfluorescein [5(6)-CF] in BC indicates that the BC structure was maintained. In cultures without ENT, 5(6)-CF was localized in BC at *t*=48 h. However, the 5(6)-CF expression signal in BC was weak at *t*=48 h in the culture with ENT. From these results, it is clear that the dynamic behavior of BC resulted in the alteration of hepatic function and BC structure.

### Exposure to forskolin (FOR) or sodium taurocholate (TAU) allows robust BC structure

**Fig. 3.**
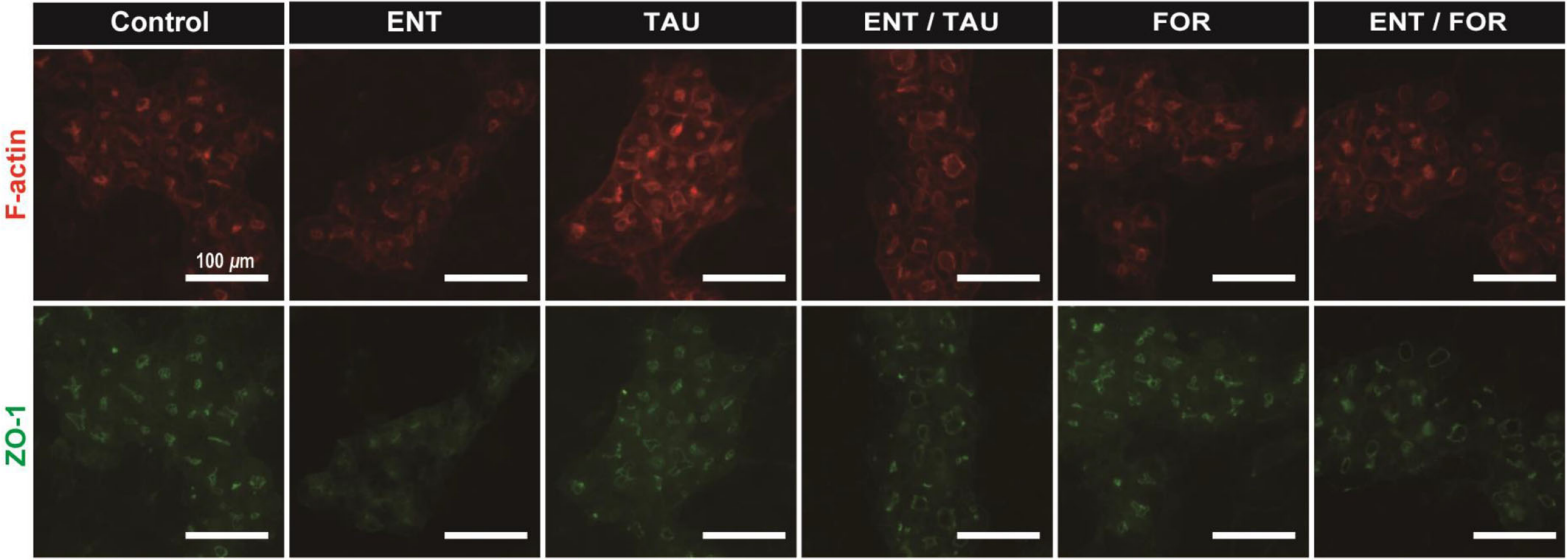
**Exposure of HepaRG cells cultured with ENT to TAU or FOR inhibits the disruption of TJs through BC dilation.** Immunofluorescence images of F-actin (Phalloidin, red) and TJ protein (ZO-1, green) for HepaRG cells cultured without drugs and those cultured with ENT (100 µM), TAU (100 µM), ENT/TAU (100/100 µM), FOR (10 µM), and ENT/FOR (100/10 µM) at *t*=48 h. The immunofluorescence images were captured using fluorescence microscope with a 10× objective lens. The images are representative of more than three independent experiments that gave similar results. Scale bars, 100 μm.

Based on our findings, we hypothesized that a robust BC structure protects against cell death in the drug-induced intrahapetic cholestasis process. To test this hypothesis, we used FOR or TAU, which is a drug that facilitates TJ formation via the cAMP-Epac-MEK-AMPK pathway (Fu et al., 2011). Exposure to ENT (100 µM), TAU (100 µM), FOR (10 µM), ENT/TAU (100/100 µM), and ENT/FOR (100/10 µM) was carried out in HepaRG cells 14 days after cell seeding, and the cells were fixed and stained for ZO-1 and F-actin at *t=*48 h. In cultures without drugs, ZO-1 was localized in BC in HepaRG cells, and the cells had a distinct line that surrounded the BC perfectly (Fig. 3). However, ZO-1 expression in the BC of HepaRG cells in the culture with ENT was weak (Fig. 3). Exposure of HepaRG cells to TAU or FOR in the culture with ENT allows the localization of ZO-1 in BC, robustizing BC structure (Fig. 3). Interestingly, in the cultures with ENT/FOR, ENT/TAU, and FOR, almost all cells had a distinct line that surrounded the BC, as observed in the culture without drugs, in spite of its BC dilation (Fig. 3).

For a comparison between BC dynamics and TJ structures, a time-lapse observation was conducted in HepaRG cells cultured without drugs and those cultured with ENT, FOR, and ENT/FOR at *t*=0–48 h. We carried out ZO-1 immunostaining after the time-lapse observation. There was no dynamic change in the BC in the culture with FOR at *t*=0–48 h, and the ZO-1 expression of BC in the cells was positive, similar to that in the culture without drugs (Movie 2). The cells in the culture with ENT exhibited dilation of BC, resulting in cell death. A comparison between the time-lapse and the ZO-1 staining images of cells cultured with ENT or ENT/FOR revealed that exposure to ENT and FOR resulted in the maintenance of the expanded size of BC, keeping the localization of ZO-1 in the membranes of BC (Movie 3). It is suggested that exposure to ENT/FOR suppressed the alteration of TJ localization in BC in accordance with BC dynamics.

### Suppressed change in BC dynamics protects against cell death

**Fig. 4.**
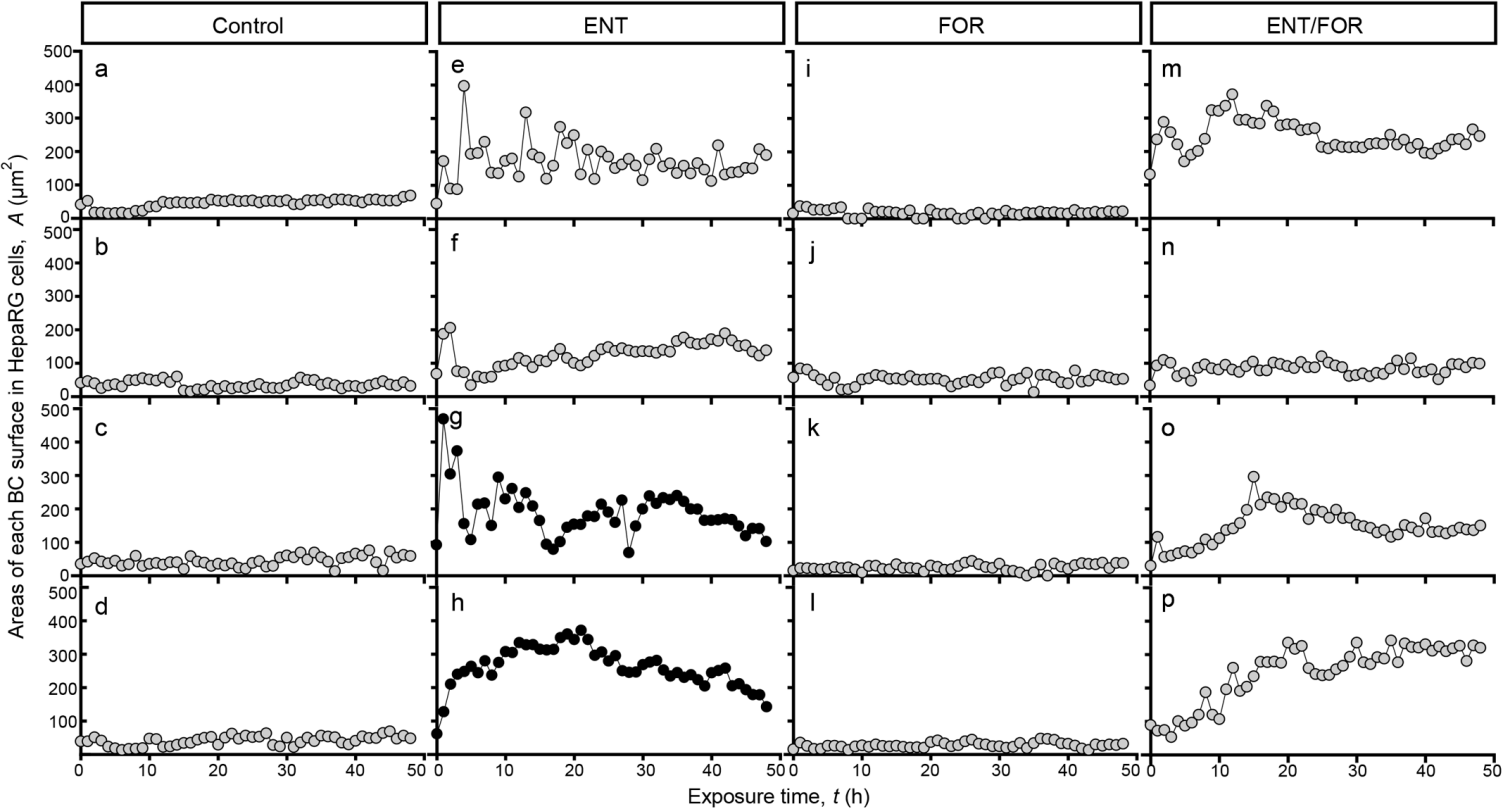
**Suppressed change in BC surface size allows for the stabilization of TJs in BC.** Representative time-lapse alterations of the area of the BC surface, which we named as *A*, in cultures without drugs (a–d) and in those cultured with entacapone (ENT; e–h, 100 µM), forskolin (FOR; i–l, 10 µM), and ENT/FOR (m–p, 100/10 µM). Retrospective analysis of ZO-1-positive and ZO-1-negative BC was conducted using the ZO-1 immunostaining images in the same region where the surface areas of BC were measured. Shaded circles and closed circles show time-dependent changes in area of ZO-1-positive and ZO-1-negative BC, respectively.

To quantitatively understand the TJ structure as a consequence of the changes in BC behaviors, we carried out a retrospective analysis of ZO-1-positive and ZO-1-negative BC against the areas of HepaRG cells cultured without drugs, those cultured with ENT, with FOR, and with ENT/FOR at *t=*0–48 h. In cells cultured without drugs, the *A* values ranged from 0 to 100 μm^2^, indicating a suppressed change in the size of BC (Fig. 4A–D). Conversely, the cells in the culture with ENT showed a rapid increase in the *A* values until *t=*6 h, and the values exhibited the dynamic change in the size of BC at *t=*0–48 h (Fig. 4E–H). In the culture with ENT/FOR, the cells showed a gradual increase in the *A* values until *t=*24 h, and exhibited suppressed changes in the size of BC at *t=*24–48 h (Fig. 4M–P). In all culture conditions, the ZO-1-positive BC showed a suppressed amplitude of change in BC size, whereas the ZO-1-negative BC showed a drastic change in the size of BC (Fig. 4).

**Fig. 5.**
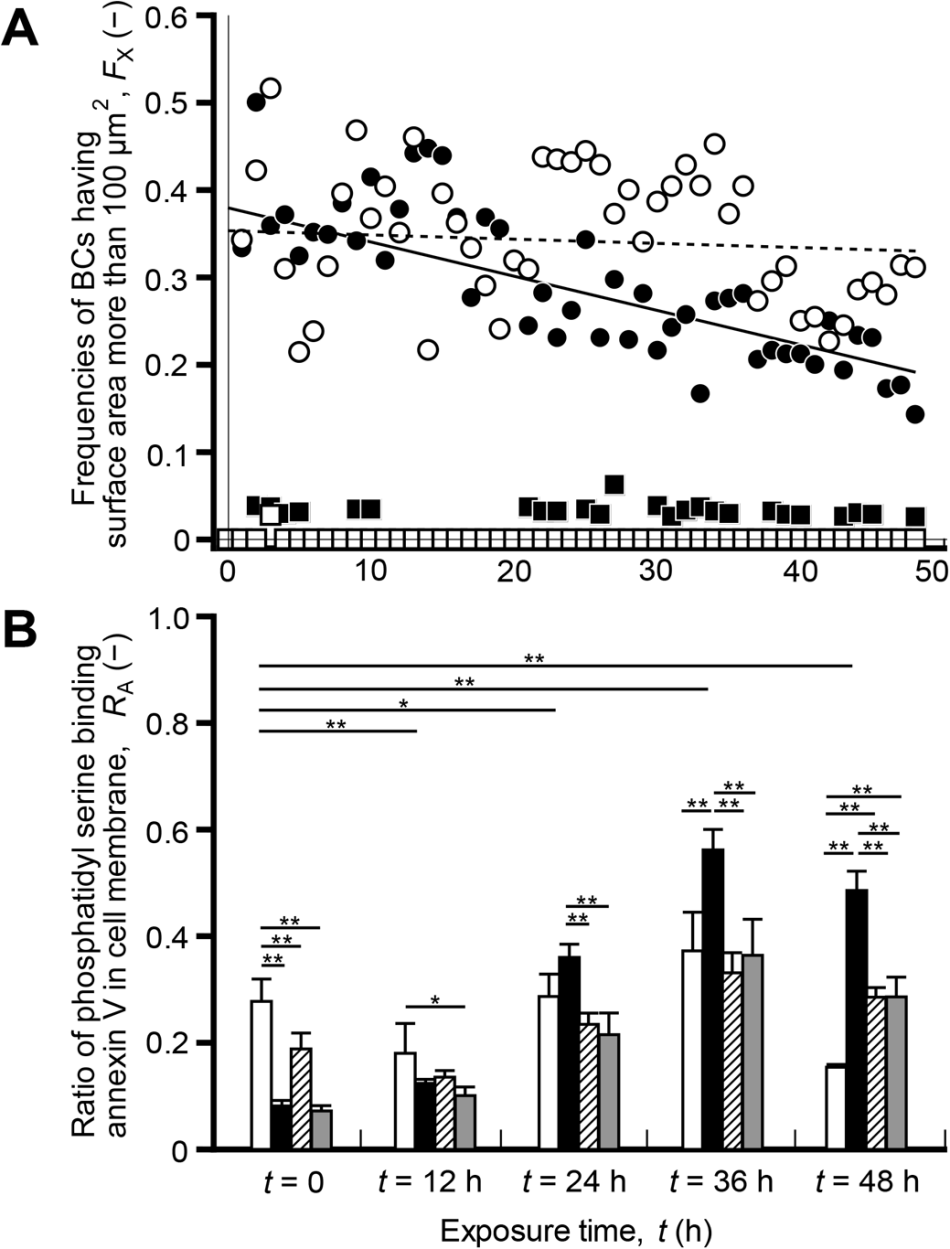
**Exposure of HepaRG cells cultured with ENT (100 µM) to FOR** (**10 µM) induces the maintenance of BC size and the suppression of apoptosis.** (A) Frequencies of BC with surface areas more than 100 µm^2^, *F*_X_, in cells cultured without drugs and in those cultured with ENT (100 µM), FOR (10 µM), ENT/FOR (100/10 µM). The phase-contrast images were captured every 20 min for 48 h, and the data were obtained from 30–60 BCs of HepaRG cells in the region of interest (ROI, 300×300 µm). Total number of BCs and the number of BCs having surface area more than 100 μm^2^ were counted every 1 h in HepaRG cells cultured without drugs, those cultured with ENT, with FOR, and with ENT/FOR. The *F*_X_ were calculated as the ratio of BCs having surface area more than 100 μm^2^ to the total number of BCs. Opened square, closed circles, closed square, and opened circles show the *F*_X_ values of the cells in the culture without drugs and those cultured with ENT, FOR, ENT/FOR, respectively. The solid and broken lines were obtained by linear regression of *F*_X_ of ENT and ENT/FOR, respectively (Igor Pro; Wavemetrics, OR, USA). (B) Ratio of phosphatidylserine binding annexin V in cell membrane, *R*_A_, in culture without drugs, those cultured with ENT (100 µM), FOR (10 µM), ENT (100 µM) and FOR (10 µM) at *t*=0, 12, 24, 36, 48 h. Opened bars, closed bars, hatched bars, and shaded bars show *R*_A_ values in the culture without drugs and those in cultures with ENT (100 µM), FOR (10 µM), ENT/FOR (100/10 µM), respectively. In the apoptotic process, the localization of phosphatidylserine shows the change from the inner to outer leaflet of the cell membrane, annexin V binds to the phosphatidylserine on the outer membrane. Using RealTime-Glo™ Annexin V Apoptosis and Necrosis Assay, the apoptotic cells were measured by the exposure of phosphatidylserine on the outer leaflet of the cell membrane during the apoptotic process, and assay signals were detected using a plate-based multimode reader. The maximum *R*_A_ value, i.e., positive control, was determined based on the data of HepaRG cells cultured with raptinal (10 µM), which is an apoptosis inducer. Data were obtained from six independent experiments.

From these results, we noticed that the change in the size of BC may be an important event that governs cell death during drug-induced intrahepatic cholestasis. Thus, to confirm this notion, the frequency of BC with a surface area greater than 100 μm^2^, *F*_X_, and ratio of phosphatidyl serine binding annexin V in the cell membrane, *R*_A_, were investigated at *t=*0–48 h. In cells cultured without drugs, the *F*_X_ values were less than 0.1, and there was no change in the *F*_X_ values at *t*=0–48 h (Fig. 5A). However, the maximum *F*_X_ values of the cells cultured with ENT or ENT/FOR were almost identical, which is 0.50 and 0.52, respectively. The cells cultured with ENT showed a decrease in *F*_X_ values after the initial increase in *F*_X_ values, but exposure to ENT/FOR showed a lesser decrease in *F*_X_ values compared with that under the ENT culture condition (Fig. 5A). The *R*_A_ values in the culture with ENT increased significantly with decreasing *F*_X_ values at *t*=0–48 h (Fig. 5A,B). Conversely, the cells cultured with ENT/FOR exhibited constant *F*_X_ values and significantly suppressed apoptosis at *t*=0–48 h (Fig. 5A,B). The exposure of HepaRG cells to FOR in the culture with ENT was found to suppress the alteration of the BC size through the robust BC structure, resulting in the inhibition of apoptosis.

## DISCUSSION

**Fig. 6.**
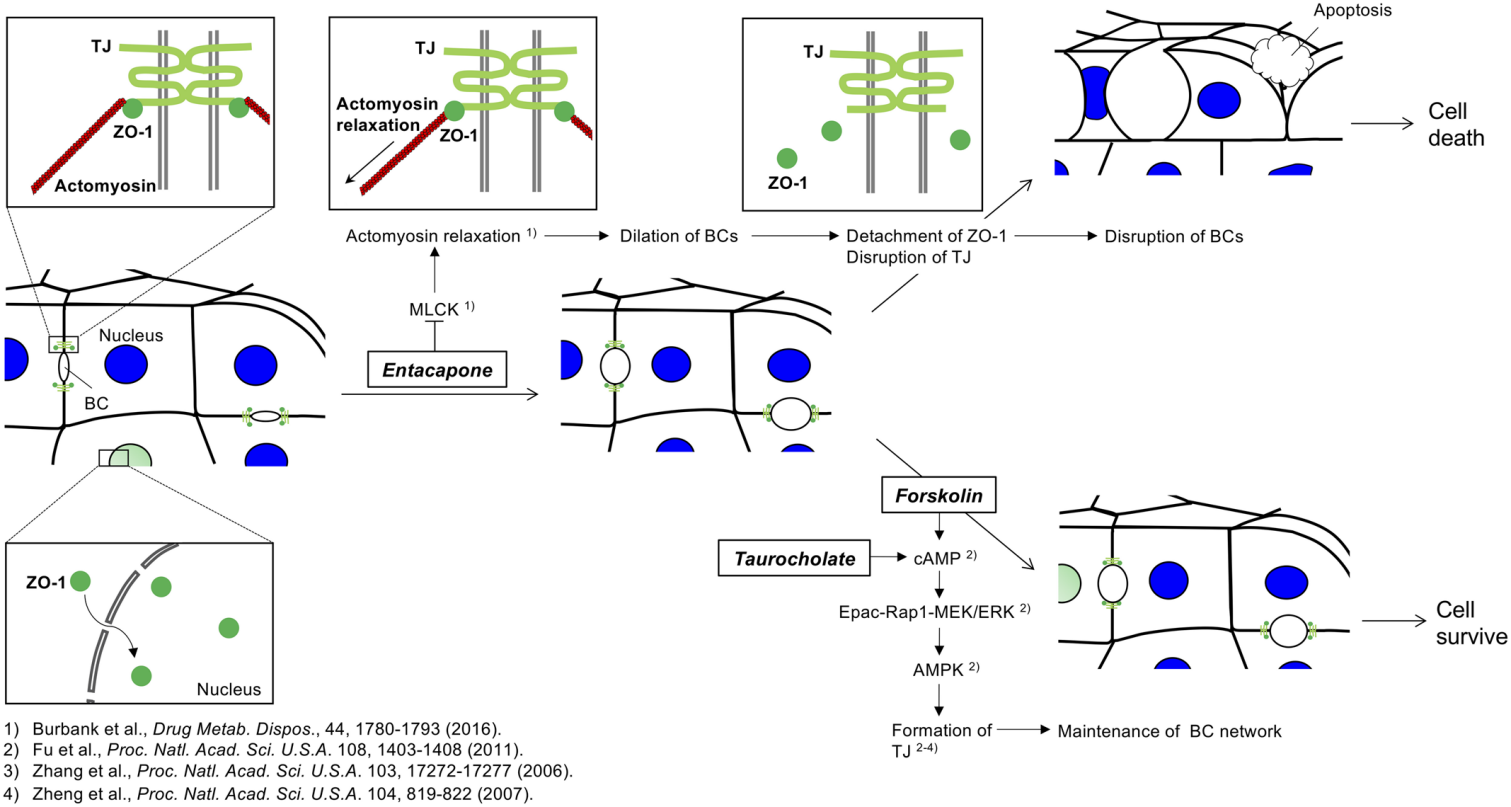
**Stabilization of TJs protects against HepaRG cell death during drug-induced intrahepatic cholestasis.** Schematic diagram of cell fate through the structural changes in accordance with BC dynamics in HepaRG cells during drug-induced intrahepatic cholestasis. See discussion for further explanation.

Our study has revealed that suppressed BC dynamics cause the stabilization of TJ, leading to the inhibition of apoptosis. We suggest that TJ structural changes in accordance with BC dynamics affect the fate of HepaRG cells during drug-induced intrahepatic cholestasis. As shown in Fig. 6, the stabilization of TJ in BC is considered to suppress apoptosis of HepaRG cells during drug-induced intrahepatic cholestasis. Mature HepaRG cells express TJ proteins (claudin and occludin) in the cellular membrane, which separates the apical and basolateral membrane domains, in BC (Wood, 1965; Montesano et al., 1975; Luzzatto, 1981). The ZO-1 protein binding to the TJ localized at the cellular membrane stabilizes BC, and the actin cytoskeleton binds to the TJ proteins via ZO-1. Moreover, ZO-1 exhibits several nuclear localization and nuclear export signals, enabling it to shuttle between the cytoplasm and the nucleus (Itoh et al., 1997; Fanning et al., 1998), and the ZO-1 of dual localization at the cell nucleus and membrane predominantly regulates cell growth and polarity, respectively (Balda and Matter, 2000; Balda et al., 2003; Bauer et al., 2010). From these reports, in the present study, we consider that ZO-1 localization in the nucleus implies the phase of cell growth, whereas ZO-1 localization in cell membrane implies the phase of maintenance of hepatocyte polarity. When exposed to ENT, HepaRG cells exhibit rapid dilation of BC. With an increase in the size of BC, HepaRG cells exhibit the disruption of BC, associated with the detachment of ZO-1. At the same time, the cells cause the disruption of TJ shown by the disappearance of claudin-1. Loss of TJ induces hepatocyte depolarization, which causes bile secretory failure, i.e. cholestasis, leading to liver damage (Gissen and Arias, 2015). Ultimately, the cells cultured with ENT undergo apoptosis. These results indicate that cell death is due to TJ structural changes in accordance with BC dynamics in cells cultured with ENT. However, when exposed to FOR or TAU in a culture with ENT, HepaRG cells exhibit the formation of TJ and the inhibition of apoptotic cell death. This means that apoptotic cell death comes from TJ structural changes, i.e. loss of ZO-1 and claudin-1 localization, in response to BC dynamics as observed in the present study. FOR or TAU are known to significantly increase cAMP, and signaling via the cAMP-Epac-Rap1-MEK pathway activates AMP-activated protein kinase (AMPK) (Fu et al., 2011). AMPK regulates TJ assembly (Zhang et al., 2006; Zheng and Cantley, 2007). These reports suggest that the maintenance of TJ via the cAMP-Epac-Rap1-MEK signaling pathway allows the inhibition of apoptosis in cells cultured with ENT and TAU or FOR. Thus, it can be stated that the increase in cAMP causes the stabilization of ZO-1 in BC of HepaRG cells cultured with ENT and FOR or TAU, resulting in the suppression of apoptosis.

To investigate whether the maintenance of TJ in BC suppresses cell death, we used TAU or FOR to facilitate TJ formation. Our findings clarify that the stabilization of TJ inhibited cell death due to the drastic behaviors of BC in HepaRG cells during drug-induced intrahepatic cholestasis. Some research groups have reported that TJ disruption in the BC of hepatocytes, from transgenic mice and cultured hepatocytes, results in depolarization and hepatocellular injury (Kojima et al., 1999; Sambrotta et al., 2014). Anderson et al. have demonstrated that the loss of ZO-1 leads to liver cholestasis in rats (Anderson et al., 1989). In addition, diseased liver, which loses its polarized structure, alters the gene expression of TJ proteins such as claudin-1 and ZO-2 (Hadj-Rabia et al., 2004; Sambrotta and Thompson, 2015). These reports indicate that TJ proteins are deeply involved in the maintenance of hepatocyte structure and function. Thus, we consider that exposure of HepaRG cells to ENT induced apoptosis due to TJ disruption via drastic BC dynamics. Interestingly, exposure to TAU or FOR in the culture with ENT inhibited apoptosis in the present study. Mayati et al. have reported that FOR facilitates TJ formation and hepatic polarization in BC of HepaRG cells during the transition phase from immature to mature state (Mayati et al., 2018). Fu et al. have reported that TAU or FOR significantly increases the amount of cAMP and facilitate hepatocyte polarization as well as TJ formation (Fu et al., 2011). Based on these findings, it is most likely that FOR or TAU maintained the TJs in BC, resulting in a robust BC structure and the suppression of apoptosis in the present study.

Most studies have focused on the influence of drugs on the function of hepatic transporters and enzymes related to mitochondrial toxicity. It has been shown that the inhibition of hepatic transporters leads to toxic concentrations of bile acids in the cytoplasm, causing mitochondrial impairment (Pedersen et al., 2013; Qui et al., 2016; Rodrigues et al., 2018). Grünig et al. have reported that ENT uncouples oxidative phosphorylation and inhibits mitochondrial enzyme complexes (Grünig et al., 2017). These reports suggest that loss of transporter function may induce liver injury due to mitochondrial impairment. However, it has been reported that ENT does not directly alter bile salt export pump activity when compared with other cholestasis drugs such as bosentan and troglitazone (Burbank et al., 2016). From these reports, we consider that drug-induced intrahepatic cholestasis may involve other factors. In the present study, the stabilization of TJ at BCs inhibited cell death in the culture with ENT and FOR or TAU, and thus, we consider that BC disruption is a factor of cell death other than transporter inhibition.

Retrospective analysis based on time-lapse and staining images provided important information for understanding the TJ structural changes that occur as a consequence of BC dilation. We focused on the influence of TJ structural changes in accordance with BC dynamics on cell death and attempted to unveil the relationship between TJ structure and BC dynamics. In the present study, it was found that ZO-1-negative BC showed drastic changes in the size of BC, inducing BC disruption and apoptosis. Conversely, ZO-1-positive BC showed a gradual change in the size of BC under all culture conditions. HepaRG cells cause TJ disruption and apoptosis through dynamic changes in BC size. Furthermore, we demonstrated that the change in BC size is an important event that affects cell death during drug-induced intrahepatic cholestasis. We consider that the magnitude and speed of change in BC may be a crucial parameter that will further our understanding of the early phase before cell death in the drug-induced intrahepatic cholestasis process.

In conclusion, time-lapse observation and ZO-1 immunostaining in the same region were performed to understand the association between the TJ structure and BC dynamics of HepaRG cells during drug-induced intrahepatic cholestasis. The retrospective analysis based on the datasets based on time-lapse and ZO-1 immunostaining images revealed that BC dynamics are important events governing cell death during drug-induced intrahepatic cholestasis. Simultaneously, our findings demonstrate that the stabilization of TJ, using FOR or TAU, suppressed BC disruption and apoptosis.

## MATERIALS AND METHODS

### HepaRG cell culture

Cryopreserved differentiated HepaRG cells (Lot number HPR116293-TA08; Biopedric International, Saint Gregoire, France) were seeded at 2.1×10^5^ cells/cm^2^ in six-well plates (Corning, NY, USA) and maintained for 14 d at 37°C in a humidified 5% CO_2_ incubator; the medium was changed every 2 days. After 14 days, HepaRG cells showing TJ formation, which was termed the ‘mature state’ in the present study, were incubated for 48 h in a culture medium containing 1.7% dimethyl sulfoxide (DMSO) with ENT (100 μM; Sigma-Aldrich, MO, USA), TAU (100 μM; FUJIFILM Wako Pure Chemical Corporation, Osaka, Japan), FOR (10 μM; FUJIFILM Wako Pure Chemical Corporation), or without those drugs at 37°C in a humidified 5% CO_2_ environment. Exposure time, *t*, was determined from the moment of the addition of either ENT, TAU, or FOR.

### Visualization of functional bile flow in BC

To understand the transporter function of BC in HepaRG cells, we used 5(6)-CFDA (Lambda Probes and Diagnostics, Graz, Austria) to probe the functional integrity of BC. This reagent is hydrolyzed to 5(6)-CF, secreted from hepatocytes, and exhibits distinct fluorescence in BC. Mature HepaRG cells at 14 days were incubated for 48 h with and without ENT in culture medium containing 1.7% DMSO at 37°C in a humidified 5% CO_2_ environment. At *t*=48 h, the HepaRG cells in both culture conditions were washed with PBS (Nacalai Tesque, Kyoto, Japan). Then, 5(6)-CFDA was added to each well at a concentration of 10 μg/mL, and the treated HepaRG cells were incubated for 15 min at 37°C in a humidified 5% CO_2_ environment. After washing with PBS, HepaRG cells were added to fresh culture media, and the cells were captured using an image analyzer with a 10× objective lens (Eclipse Ti2; Nikon, Tokyo, Japan). The fluorescence signal intensities were obtained by excitation at the corresponding wavelength of 488 nm.

### Immunofluorescence staining

Fluorescence staining of ZO-1 protein (ab96587, Abcam, Cambridge, UK), claudin-1 (ab211737, Abcam), F-actin (Alexa Fluor 594 Phalloidin, Invitrogen, CA, USA), and cell nuclei (4′,6-diamidino-2-phenylindole, DAPI; Life Technologies Corporation, CA, USA) of HepaRG cells was performed to understand the structural changes in BC. HepaRG cells washed with PBS after the time-lapse observation were fixed with 2% paraformaldehyde for 15 min at 4°C, and permeabilized by incubation for 30 min in 0.05% Triton X-100 at 4°C. Nonspecific binding of antibodies was blocked by treatment with Block Ace (Dainippon Sumitomo Pharma, Osaka, Japan) overnight at 4°C. Primary antibodies (1:200 dilution in PBS) were incubated overnight at 4°C. After washing with PBS, HepaRG cells were incubated with Alexa Fluor 488-conjugated goat anti-rabbit IgG (Abcam), DAPI, and Alexa Fluor 594 phalloidin for 3 h at room temperature. Fluorescence images were captured using a fluorescence microscope with a 10× Eclipse Ti2 objective lens or a confocal laser microscope with a 60× objective lens (Eclipse Ti-E; Nikon, Tokyo, Japan). The fluorescence signal intensities were obtained by excitation at corresponding wavelengths of 358, 488, and 594 nm.

### Measurement of BC surface areas

The schematic outline in Fig. S1 shows the process of data analysis to quantitatively understand TJ structures as a consequence of the BC dynamics in HepaRG cells during drug-induced intrahepatic cholestasis. Time-lapse images of HepaRG cells were captured using an image analyzer with a 10× Eclipse Ti2 objective lens. Original images (5.3×5.3 mm) at 16 bits in grayscale were captured every 20 min for 48 h from triplicate cultures in six-well plates. To determine the areas of the BC surface in HepaRG cells, the region of interest (ROI; 300×300 μm) was defined (Fig. S1B). Areas of the BC surface, *A*, from the phase-contrast image datasets were measured manually using image software (NIS-Elements software, Nikon, Tokyo, Japan). The data from areas of BC were obtained from 30–60 BCs within ROIs of HepaRG cells in each culture condition.

To ascertain and quantify the association between TJ structure and BC dynamics, ZO-1 immunostaining images were captured in the same positions where areas of BCs were measured previously. The BC of HepaRG cells were divided into two groups, namely, ZO-1-positive and ZO-1-negative groups, according to the criteria depicted in Fig. S1C. The ZO-1-positive and ZO-1-negative BCs were retrospectively identified, and their estimated frequencies (*F*_Z_ and *F*_N_, respectively) are presented in Fig. S1D.

### Apoptosis assay

For the apoptotic assay, HepaRG cells were seeded at 2.1×10^5^ cells/cm^2^ in 96-well plates, which are polystyrene plate frames with white pigments and transparent polystyrene bottoms (ViewPlate-96 TC; PerkinElmer, MA, USA). We checked cell morphology using phase-contrast microscopy and confirmed that there was no difference in the extent of mature states between the six- and 96-well plates. To minimize luminescence crosstalk between wells, the white adhesive bottom seal was attached to the transparent polystyrene plates, turning the bottom of the microplate opaque. After 14 days of cell seeding, mature HepaRG cells were incubated for 48 h in culture medium containing 1.7% DMSO without drugs and in medium containing either ENT (100 μM), TAU (100 μM), or FOR (10 μM), or ENT/FOR (100/10 μM) at 37°C in a humidified 5% CO_2_ environment. The apoptotic assay was conducted using a RealTime-Glo™ Annexin V Apoptosis and Necrosis Assay Kit (Promega, WI, USA) according to the manufacturer's protocol, and the apoptotic assay signal was detected using a multi-detection microplate reader (Synergy™ HT, BioTek, VT, USA) every 12 h. The localization of phosphatidylserine in apoptotic cells shows a shift from the inner to outer leaflet of the cell membrane, and annexin V binds to phosphatidylserine on the outer membrane. This assay kit was used to measure the exposure of phosphatidylserine on the outer leaflet of the cell membrane during the apoptotic process, leading to a quantitative and real-time understanding of apoptosis-induced cells. The ratio of phosphatidylserine binding to annexin V in the cell membrane, *R*_A_, was calculated using the following equation:
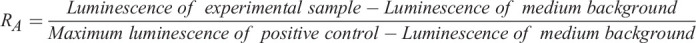
The maximum *R*_A_ value was determined based on the data of HepaRG cells cultured with apoptosis-inducer raptinal (10 μM; Sigma-Aldrich) (Palchaudhuri et al., 2015), which was used as a positive control.

### Statistical analysis

Data were expressed as means with standard deviations, and comparisons between the groups were determined by one-way analysis of variance and the Tukey–Kramer post-hoc test. For all analyses, *P*-values are represented as follows: *P<0.05, **P<0.01.

## Supplementary Material

Supplementary information
