# Supplementary material for: Tight junction stabilization prevents HepaRG cell death in drug-induced intrahepatic cholestasis
Source: Biol Open. 2021 Jun 22;10(6):bio058606. doi: 10.1242/bio.058606 (PMC8272035; doi:10.1242/bio.058606)
Supplement: Supplementary information [file biolopen-10-058606-s1.pdf]

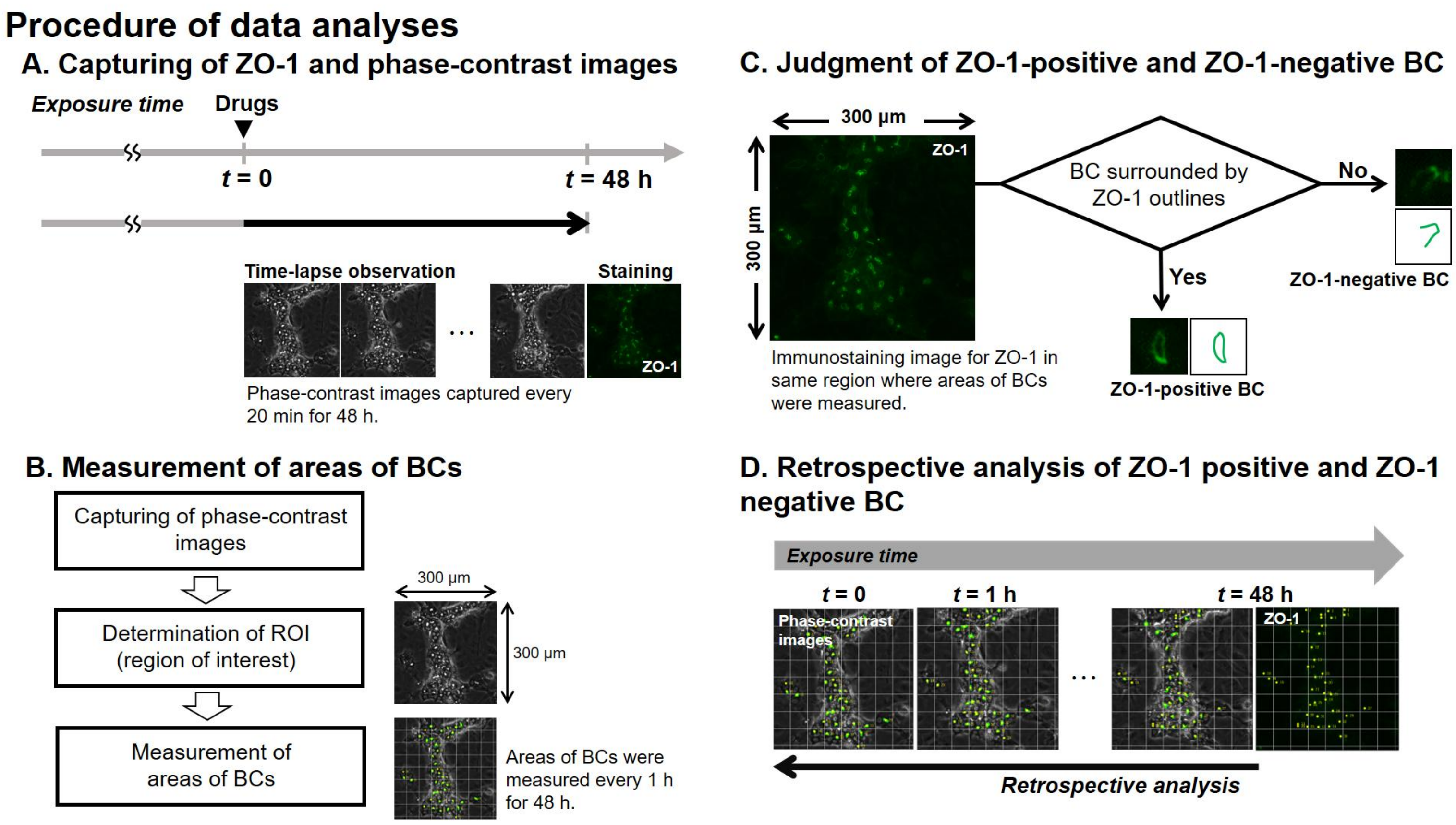

Figure S1 Procedure for retrospective analysis based on time-lapse observations and immunofluorescence images of HepaRG cells during drug-induced intrahepatic cholestasis. BC = bile canaliculi. *t* = exposure time.

Biology Open • Supplementary information

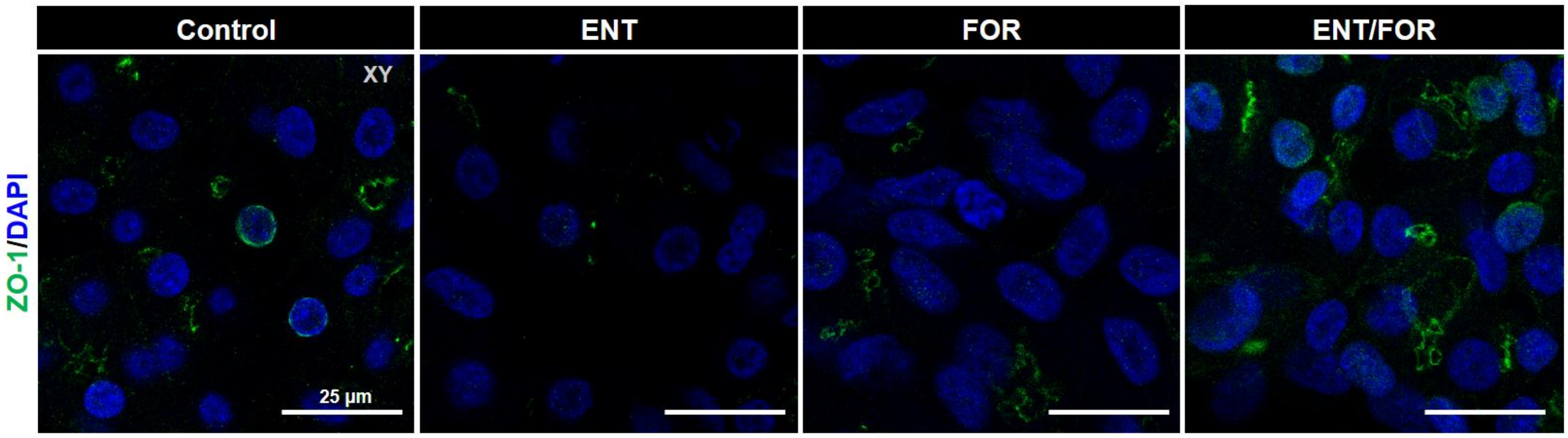

Figure S2 Confocal immunofluorescence images of cell nuclei (DAPI, blue) and tight junction (TJ) protein (ZO-1, green) for HepaRG cells cultured without drugs and those cultured with entacapone (ENT; 100 µM), forskolin (FOR; 10 µM), and ENT/FOR (100/10 µM) at $t = 48$ h. Confocal immunofluorescence images show 2-D optical cross-sectioning (XY planes). The scale bars show 25 µm. ZO-1 = zonula occludens-1.

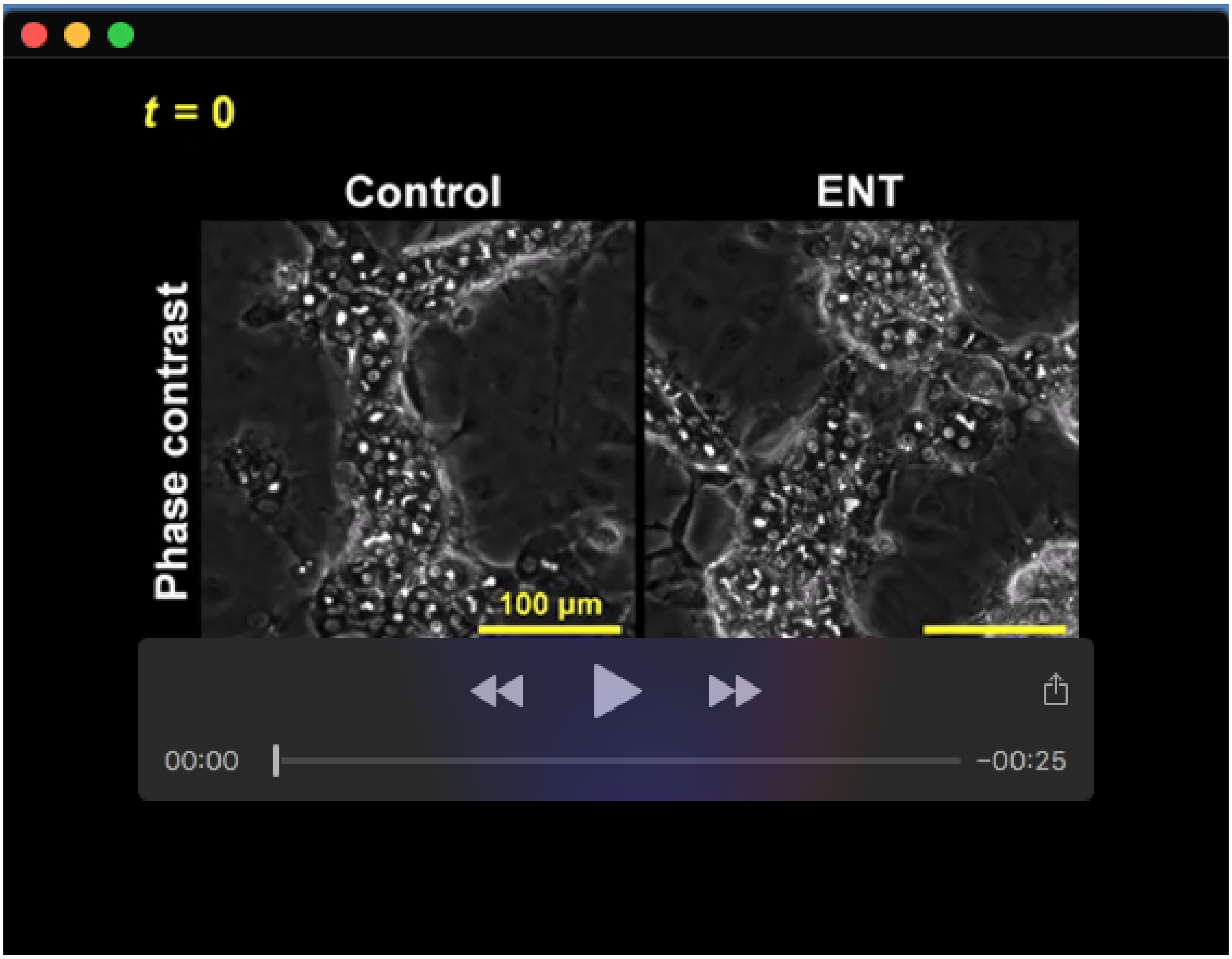

Movie 1. Structural changes in tight junctions (TJ) in accordance with bile canaliculi (BC) dynamics in HepaRG cells cultured with ENT and without at t=0–48 h.

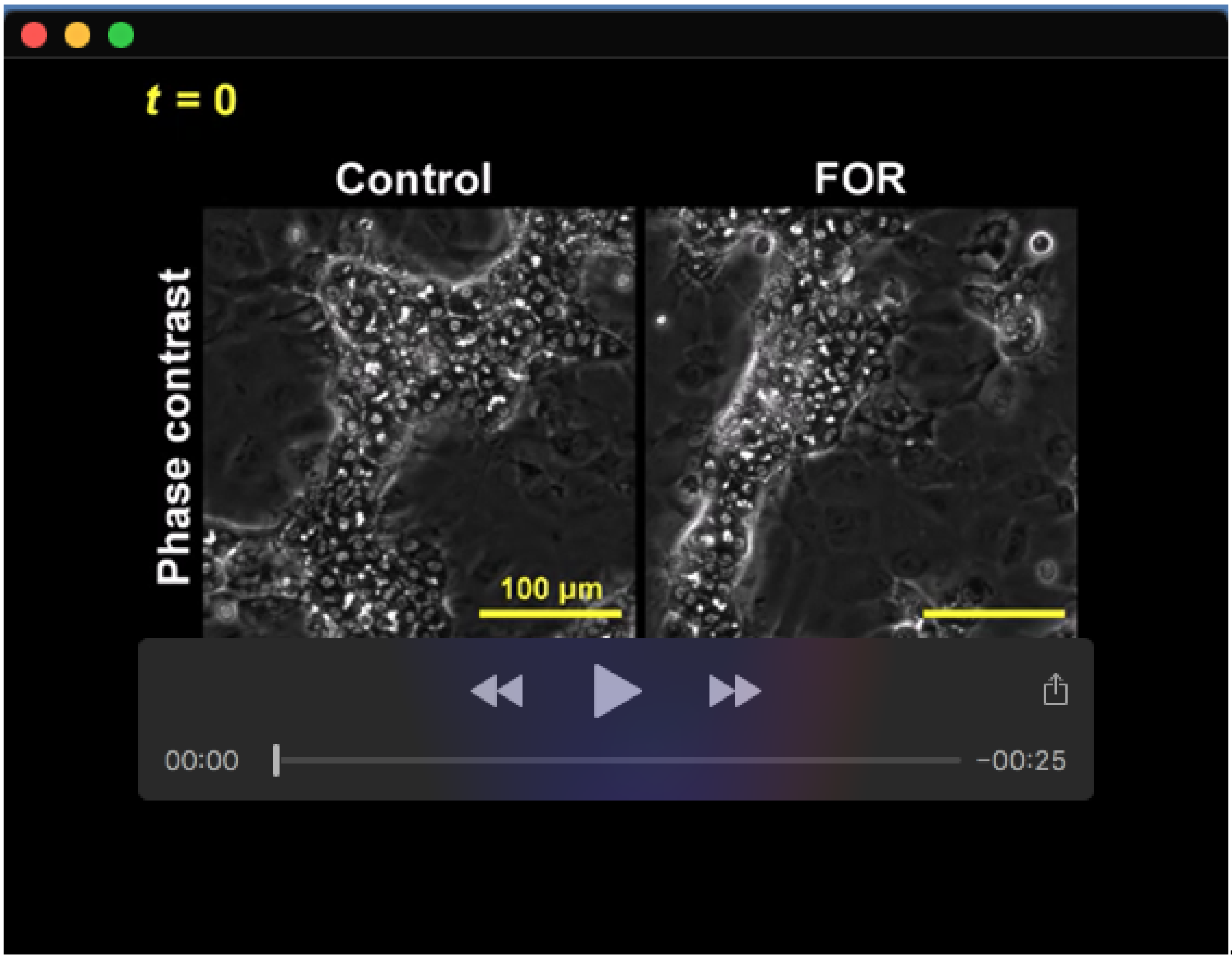

Movie 2. Structural changes in tight junctions (TJ) in accordance with bile canaliculi (BC) dynamics in HepaRG cells cultured with FOR and without at t=0–48 h.

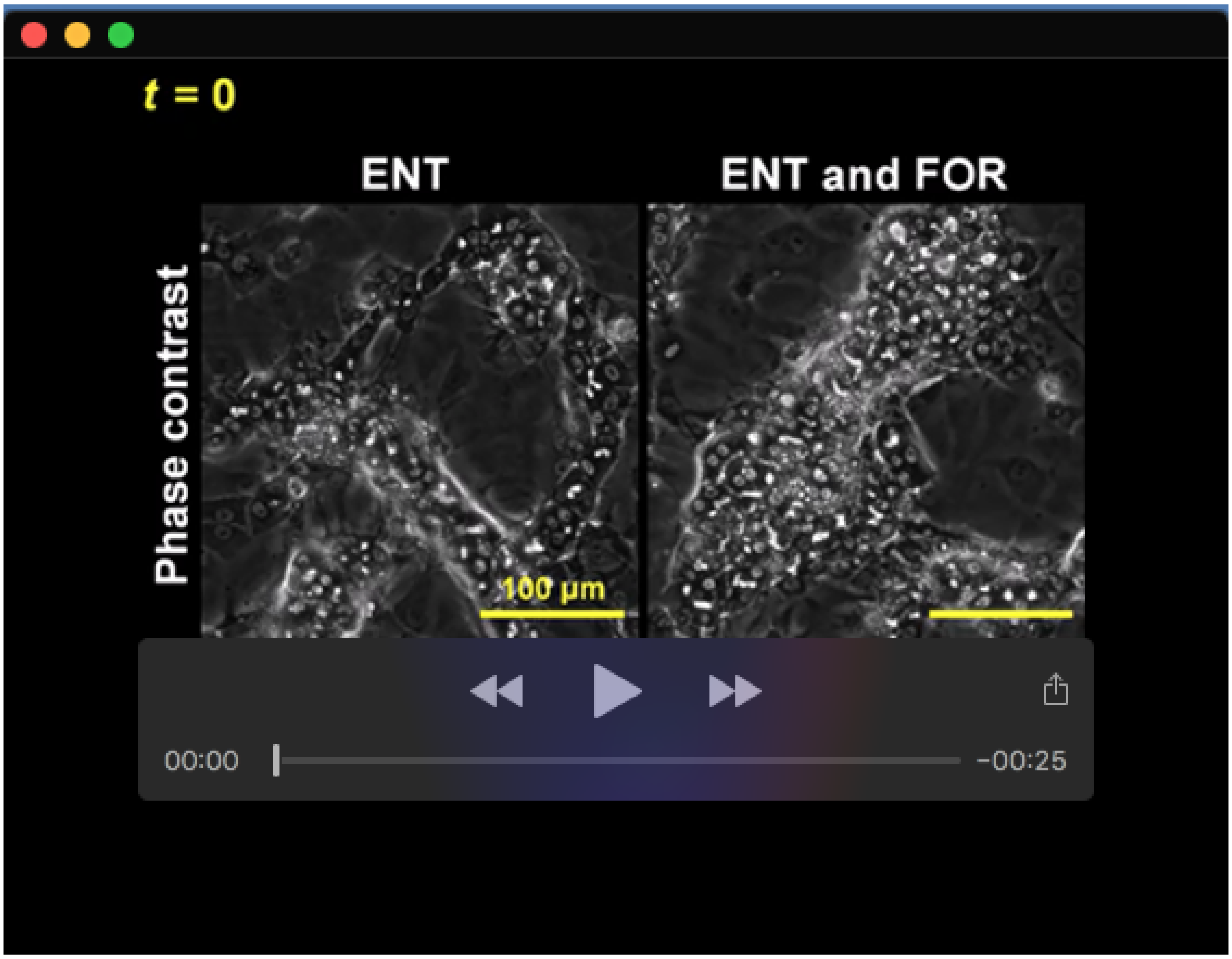

Movie 3. Structural changes in tight junctions (TJ) in accordance with bile canaliculi (BC) dynamics in HepaRG cells cultured with ENT at t=0–48 h exposed to FOR and those that are not.

Biology Open • Supplementary information
